# The EcpD Tip Adhesin of the *Escherichia coli* Common Pilus Mediates Binding of Enteropathogenic *E. coli* to Extracellular Matrix Proteins

**DOI:** 10.3390/ijms231810350

**Published:** 2022-09-08

**Authors:** Rajesh Mondal, Zeus Saldaña-Ahuactzi, Jorge Soria-Bustos, Andrew Schultz, Jorge A. Yañez-Santos, Ygnacio Martínez Laguna, María L. Cedillo-Ramírez, Jorge A. Girón

**Affiliations:** 1 ICMR-Bhopal Memorial Hospital and Research Center, Bhopal 462038, India; 2 Paul G. Allen School for Global Health, College of Veterinary Medicine, Washington State University, Pullman, WA 99164, USA; 3 Facultad de Medicina Mexicali, Universidad Autónoma de Baja California, Mexicali 21100, Mexico; 4 Department of Microbiology and Molecular Genetics, University of Florida, Gainesville, FL 32611, USA; 5 Facultad de Estomatología, Benemérita Universidad Autónoma de Puebla, Puebla 72592, Mexico; 6 Centro de Investigación en Ciencias Microbiológicas, Benemérita Universidad Autónoma de Puebla, Puebla 72592, Mexico; 7 Centro de Detección Biomolecular, Benemérita Universidad Autónoma de Puebla, Puebla 72592, Mexico

**Keywords:** ECP, fibronectin, ECM, pilus, EPEC

## Abstract

The attachment of enteropathogenic *Escherichia coli* (EPEC) to intestinal epithelial cells is facilitated by several adhesins; however, the individual host-cell receptors for pili-mediated adherence have not been fully characterized. In this study, we evaluated the hypothesis that the *E. coli* common pilus (ECP) tip adhesin protein EcpD mediates attachment of EPEC to several extracellular matrix (ECM) glycoproteins (fibronectin, laminin, collagens I and IV, and mucin). We found that the Δ*ecpA* mutant, which lacks production of the EcpA filament but retains EcpD on the surface, adhered to these glycoproteins below the wild-type levels, while the Δ*ecpD* mutant, which does not display EcpA or EcpD, bound significantly less to these host glycoproteins. In agreement, a purified recombinant EcpD subunit bound significantly more than EcpA to laminin, fibronectin, collagens I and IV, and mucin in a dose-dependent manner. These are compelling data that strongly suggest that ECP-producing EPEC may bind to host ECM glycoproteins and mucins through the tip adhesin protein EcpD. This study highlights the versatility of EPEC to bind to different host proteins and suggests that the interaction of ECP with the host’s ECM glycoproteins may facilitate colonization of the intestinal mucosal epithelium.

## 1. Introduction

The process of causing disease by bacterial pathogens includes multiple events involving the interaction of surface adhesin proteins with host cells. Enteropathogenic *Escherichia coli* (EPEC) strains, which cause infantile diarrhea in developing countries, bind to small bowel epithelial cells, forming characteristic localized adherence microcolonies due to the production of the bundle-forming pilus (BFP) [1,2]. Colonization of the small bowel results in a distinct histopathology characterized by the production of attaching and effacing (AE) lesions attributed to the genes encoded in the pathogenicity island called the locus of enterocyte effacement (LEE) [3]. The molecular mechanism by which EPEC binds and affects enterocytes is considered a multistage process. EPEC uses fimbrial and nonfimbrial adhesins to adhere to host cells, including an outer membrane protein called intimin that binds to its translocated intimin receptor (Tir) [4]. Interestingly, it has been demonstrated that EPEC can extract nutrients from infected host cells through alternative nanotubes assembled via the inner membrane components of the type III secretion system (T3SS) [5]. Furthermore, some T3SS effectors involved in bacterial colonization can hijack host endosomes at specific bacterial adherence sites, facilitating the recycling and endocytosis of plasma membrane proteins, allowing their enrichment and mislocalization at specific infection sites [6]. Upon interaction of the effectors with their intracellular targets, signal transduction cascades are initiated, resulting in cytoskeleton reorganization and formation of a pedestal under the attaching bacterium, rupture of tight junctions, and increased permeability, which results in fatal watery diarrhea in infants [7].

In addition to the BFP- and intimin–Tir-mediated interactions, other adhesins with a synergistic role in EPEC adherence include curli and flagella [1,8,9,10]. Also, the *E. coli* common pilus (ECP) produced by fecal *E. coli* and by pathogenic *E. coli* has been demonstrated to mediate adherence to cultured epithelial cells or animal gut explants and promote biofilm formation. BFP and ECP have been shown to act synergistically to facilitate host cell attachment [10]. The ECP is a long filament (3–5 nm in diameter) composed of the major pilin subunit EcpA, at the tip of which sits EcpD, a 60 kDa protein that also polymerizes to form a microfilament [11]. The location of the EcpD fiber at the tip of the EcpA filament is conceivable with the hypothesis of it being a tip adhesin, similar to the role FimH or PapG play for the type 1 pilus or the P pilus, respectively [12]. Previous reports show that the mutation in *ecpA* yields a strain that lacks the EcpA fibers but retains the ability to display the EcpD fiber on the surface. In contrast, the Δ*ecpD* mutant lacks both EcpA and EcpD [11,13,14]. 

Many bacterial pathogens such as *Neisseria gonorrhoeae*, *Brucella abortus*, *Staphylococcus aureus*, and pathogenic *E. coli* recognize extracellular matrix (ECM) glycoproteins (fibronectin (Fn), laminin (Ln), vitronectin, and collagen (Cn)) and utilize them to colonize host tissues [15,16,17,18,19,20]. Several fimbrial adhesins of pathogenic *E. coli*, such as curli, aggregative fimbriae II (AAF-II) of enteroaggregative *E. coli*, long polar fimbriae (LPF) of enterohemorrhagic *E. coli*, have been demonstrated to bind to various ECM glycoproteins [16,17,21]. Furthermore, the flagella of EPEC O127:H6 have been demonstrated to bind to ECM glycoproteins [9]. ECM glycoproteins contribute to the processes necessary for the growth and regulation of intestinal cell homeostasis [22,23,24,25] and can act as linking molecules to other membrane molecules involved in internal signaling pathways [22]. For example, Fn and Ln possess domains that can bind to other receptors, including integrins and collagens. *S. aureus* binds to eukaryotic cells through Fn, which bridges the union between the integrin and bacteria [20,26]. A myriad of integral proteins, glycoproteins, and glycolipids can be found as cell membrane receptors in the niche of the gut lumen. The human small bowel mucosal surface is lined with a variety of ECM glycoproteins that function as structural components of epithelial cells both extending into the lumen and residing in the basement membrane [25]. The majority of ECM glycoproteins reside in the basal membrane, which is not generally exposed to intruding pathogens unless tissue injury occurs, causing ECM constituents to be accessible to bacterial adhesins facilitating microbial-associated disease [19].

The nature of the host-cell receptors recognized by ECP has not been examined. In this paper, we determined the capability of EPEC prototypic strain E2348/69 and derivative isogenic Δ*ecpA* and Δ*ecpD* mutants to bind ECM glycoproteins and mucin (Mu). In addition, purified recombinant EcpA and EcpD proteins were used to assess the binding affinity for ECM glycoproteins. We demonstrate analogous data of bacteria and purified ECP proteins binding to ECM glycoproteins. Notably, EcpD was shown to function as a pilus tip adhesin, which mediates the binding of EPEC to several basal membrane ECM glycoproteins. It is possible that the ECP mediates similar interactions of other intestinal and extraintestinal *E. coli* with ECM-rich environments.

## 2. Results

### 2.1. ECP Mediates Binding of EPEC E2348/69 to Fn, Ln, Cn Type I, and Mu

**Figure 1:**
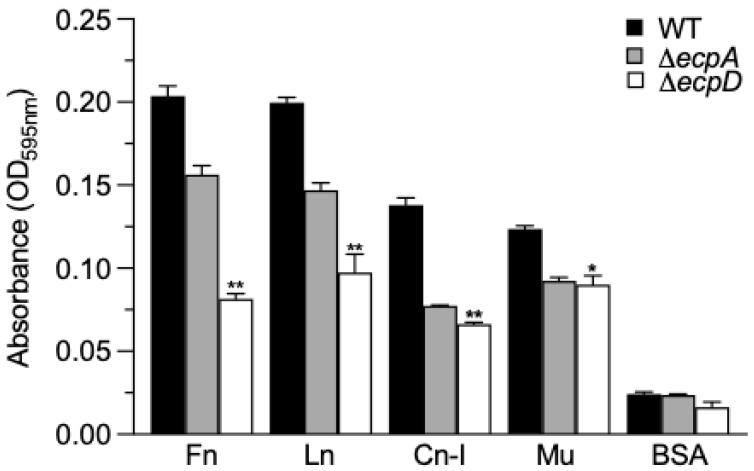
Binding of EPEC to ECM glycoproteins and Mu mediated by ECP. An ELISA-based test using anti-LPS O127 as a primary antibody was performed to quantitatively compare the binding of EPEC E2348/69 with the Δ*ecpA* and Δ*ecpD* mutant strains after 1 h of incubation at room temperature with ECM glycoproteins and Mu. BSA was used as a negative control. Data shown represent the mean of three experiments performed in triplicate on different days (Kruskal–Wallis test, * *p* < 0.05; ** *p* < 0.01).

We tested the capability of the wild-type EPEC (O127:H6) strain E2348/69 and isogenic derivative Δ*ecpA* and Δ*ecpD* mutants to bind to immobilized host ECM glycoproteins (Fn, Ln, Cn-I) and Mu at a concentration of 10 mg/mL in an ELISA-based format. These mutants were previously characterized genetically and phenotypically [11, 13, 14]. BSA was used as a binding negative control. The data show that E2348/69 binds similarly to Fn and Ln and at lower levels to Cn-I and Mu. The Δ*ecpA* mutant bound 25–30% less to Fn, Ln, and Cn-I, whereas the Δ*ecpD* mutant was more affected in terms of binding to these ECM glycoproteins. Binding to Mu was less affected in the *ecp* mutants (Figure 1). These data suggested at that point that the binding of E2348/69 to ECM glycoproteins is mediated by the tip adhesin protein EcpD.

### 2.2. The ΔecpD Mutant Yields the Lowest Binding Affinity to ECM Glycoproteins

**Figure 2:**
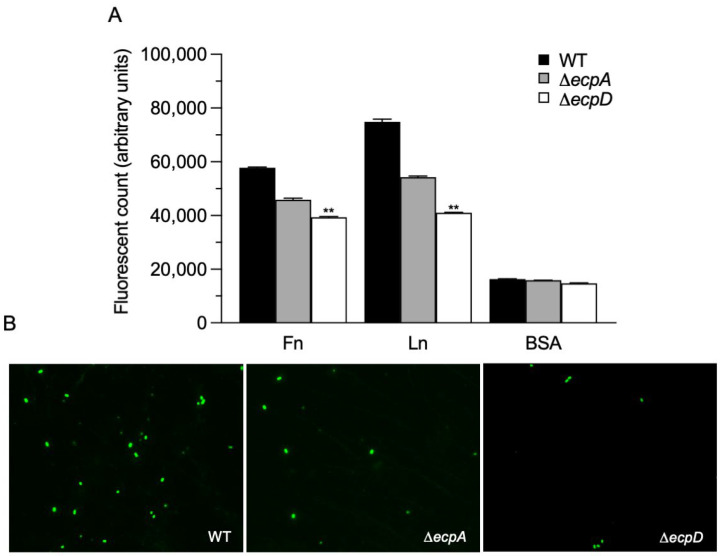
EPEC ECP-mediated binding to Fn and Ln. (**A**) A fluorescence emitter was used to quantify the binding of EPEC E2348/69 and their Δ*ecpA* and Δ*ecpD* isogenic mutants to ECM glycoproteins. Fluorescence levels were read at an OD of 595 nm. BSA was used as a negative control. (**B**) Binding of Fn with EPEC strains (green) was performed by means of immunofluorescence using anti-O127 antibodies and anti-rabbit Alexa Fluor 488. Immunofluorescence images were taken at 60×. Quantitative data shown represent the mean of three experiments performed in triplicate on different days (Kruskal–Wallis test, ** *p* < 0.01).

In the next set of experiments, we sought to confirm the binding of E2348/69 and isogenic mutants to Fn and Ln using a different approach. The binding was evaluated using a fluorescence emitter in relative fluorescence units (RFU) and then read at an OD of 595 nm. The wild-type strain E2348/69 yielded the greatest level of fluorescence to Fn and Ln, while the Δ*ecpA* mutant generated less fluorescence than the wild type, and the Δ*ecpD* mutant yielded the least fluorescence intensity in all the conditions evaluated. These data further support the proposition that EPEC-ECM glycoprotein binding is mediated by EcpD (Figure 2A). 

### 2.3. Binding of EPEC Strains to Glass Coated with ECM Glycoproteins

In addition, we performed immunofluorescence on Fn-coated glass coverslips incubated with the EPEC strains. The binding of bacteria to the glass-attached Fn was assessed with anti-O127 antibodies and anti-rabbit Alexa Fluor 488. We found that the strain that bound least to the Fn-coated surface was the Δ*ecpD* mutant, again supporting the key role of EcpD in Fn recognition (Figure 2B).

### 2.4. Confirmation of Fn Binding to EcpD by Immunoelectron Microscopy

**Figure 3:**
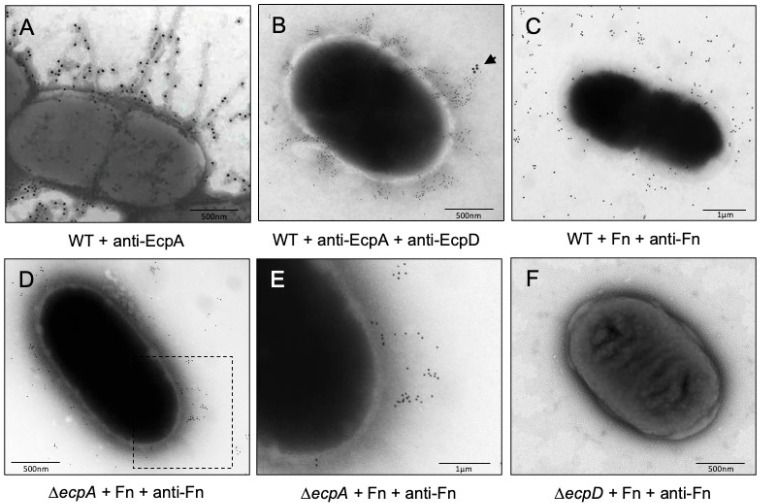
Binding of Fn to EPEC. Negative staining and immunogold labeling of E2348/69 showing production of EcpA fibers (**A**,**B**) and tip adhesin EcpD (**B**). (**C**) Binding of Fn to the wild-type strain E2348/69 using anti-Fn antibodies. (**D**,**E**) Binding of Fn to the Δ*ecpA* mutant using anti-Fn antibodies. Panel E is a magnification of the squared area shown in panel D. Note the immunostaining pattern is compatible with the reported fibrillae structure of EcpD. (**F**) No gold was present in the Δ*ecpD* mutant, which indicates no Fn binding. Arrow points to EcpD.

The EcpD protein is located at the tip of the EcpA fiber (Figure 3A,B). The Δ*ecpA* mutant does not produce the pilus stalk but it is able to display EcpD on the surface of the bacteria [11]. Hypothetically, this mutant should still be able to bind to a receptor moiety recognized by EcpD, if any. In contrast, the Δ*ecpD* mutant is an ECP-negative strain lacking the EcpA fiber and the EcpD tip. To test our hypothesis, we incubated DMEM-grown bacteria in the presence of Fn (10 mg/mL) for 1 h at room temperature. The bacteria were then stained with anti-Fn antibodies and observed by means of electron microscopy. We found that Fn bound to the wild-type strain (Figure 3C) and to the Δ*ecpA* mutant (Figure 3D,E) while no gold labeling was associated with the Δ*ecpD* mutant (Figure 3F). Collectively, the data above are compelling evidence that, in its native state, the EcpD tip protein binds to Fn. 

### 2.5. Preincubation of Bacteria with Soluble Fn Reduces Binding to Immobilized Fn

**Figure 4:**
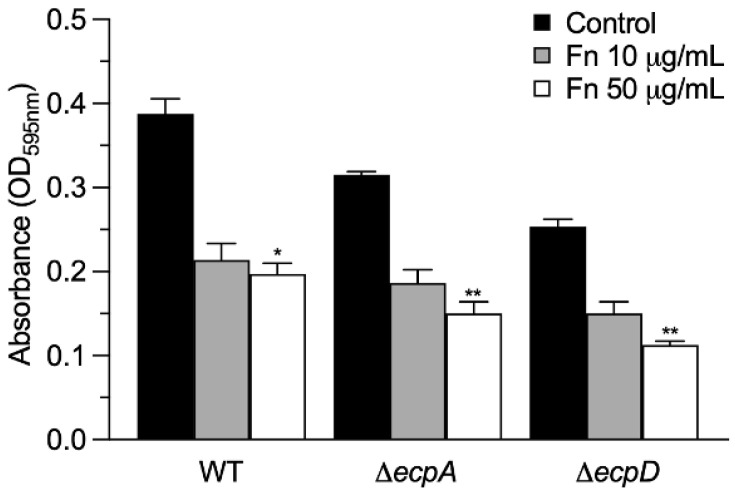
Binding of immobilized Fn to EPEC preincubated with Fn. ELISA was used to quantitatively determine the binding of immobilized Fn to EPEC strains previously incubated with soluble Fn at two different concentrations for 1 h. BSA was used as a negative control. Data are the mean of three experiments performed in triplicate (Kruskal–Wallis test, * *p* < 0.05; ** *p* < 0.01).

In this set of experiments, we wanted to know if the binding of EPEC strains to immobilized Fn, could be blocked by preincubating the bacteria with soluble Fn (at 10 and 50 mg/mL) for 1 h. We found that soluble Fn inhibited by 45% the binding of the wild-type strain to glass-attached Fn. The Δ*ecpD* mutant was blocked to a level similar to that of the Δ*ecpA* mutant (Figure 4).

### 2.6. EcpD Binds to ECM Glycoproteins with Greater Affinity Than EcpA 

**Figure 5:**
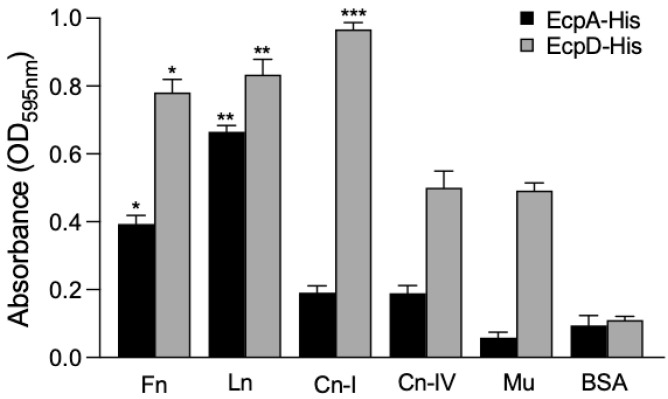
Binding of recombinant His-tagged EcpA and EcpD proteins to ECM glycoproteins and Mu. ELISA was performed to determine the binding of purified recombinant His-tagged Ecp proteins after 1 h incubation at room temperature to immobilized ECM glycoproteins and Mu. BSA was used as a negative control. Data shown are the mean of three experiments performed in triplicate on different days (Kruskal–Wallis test, * *p* < 0.05; ** *p* < 0.01; *** *p* < 0.001).

We then performed experiments of binding purified EcpA and EcpD recombinant proteins to ECM glycoproteins immobilized onto ELISA plates. Compared to EcpA, EcpD showed the highest level of binding to all the ECM glycoproteins, and the highest affinity shown was for Cn-I, Ln, and Fn (Figure 5). EcpA also bound to these glycoproteins, but with less affinity. 

### 2.7. EcpA and EcpD Bind to ECM Glycoproteins in a Dose-Dependent Manner

**Figure 6:**
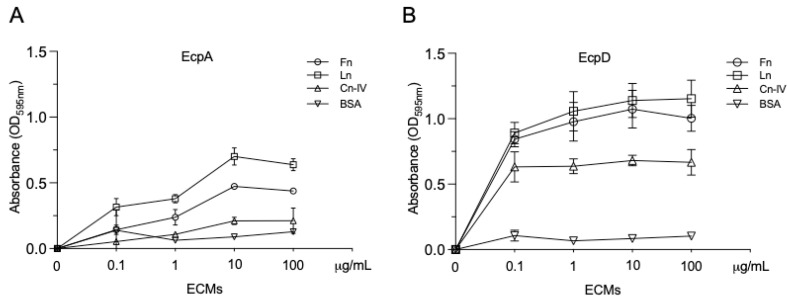
Dose-dependent binding of recombinant His-tagged EcpA and EcpD proteins to immobilized ECM glycoproteins. (**A**,**B**) Ten-fold dilutions of biotinylated Ecp proteins were added to immobilized ECM glycoproteins to determine dose-dependent binding. After washing, a streptavidin–peroxidase conjugate (1:500) was added to detect the bound biotinylated protein. BSA was used as a negative control. Data shown are the mean of three experiments performed in triplicate on different days (Mann–Whitney U test).

EcpD bound to ECM glycoproteins more than EcpA, in a dose-dependent fashion, reaching a peak at 10 mg/mL (Figure 6). Ln had the highest binding affinity, followed by Fn and Cn-IV. The same trend was observed in the other assays performed. 

### 2.8. Prior Treatment of EcpA and EcpD with Soluble Fn Inhibits Binding to Ecp Glycoproteins

**Figure 7:**
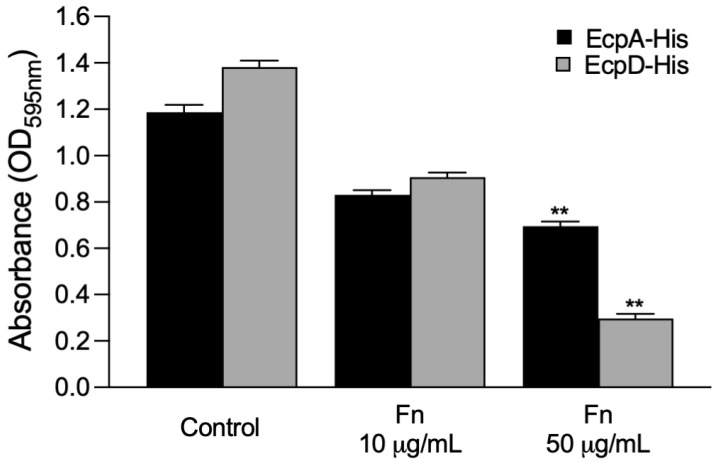
Soluble Fn inhibits binding of EcpA and EcpD to Fn. Biotinylated EcpA and EcpD proteins were incubated with 10 or 50 mg/mL of soluble Fn. The mix was then added to 96-well plates containing immobilized Fn. Binding of biotinylated proteins was detected with a streptavidin–peroxidase conjugate. BSA was used as a negative control. Data are the mean of three experiments performed in triplicate (Kruskal–Wallis test, ** *p* < 0.01).

EcpA and EcpD proteins were incubated with 10 or 50 mg/mL of soluble Fn. The mix was then added to 96-well plates containing immobilized Fn. Binding of both Ecp proteins to fixed Fn was most significantly inhibited with 50 mg/mL of Fn. There was a greater binding inhibition of EcpD than of EcpA, suggesting a greater role of EcpD in binding to this ECM glycoprotein compared to EcpA (Figure 7).

### 2.9. Pulldown Assay Demonstrates Binding of EcpA and EcpD to Fn

**Figure 8:**
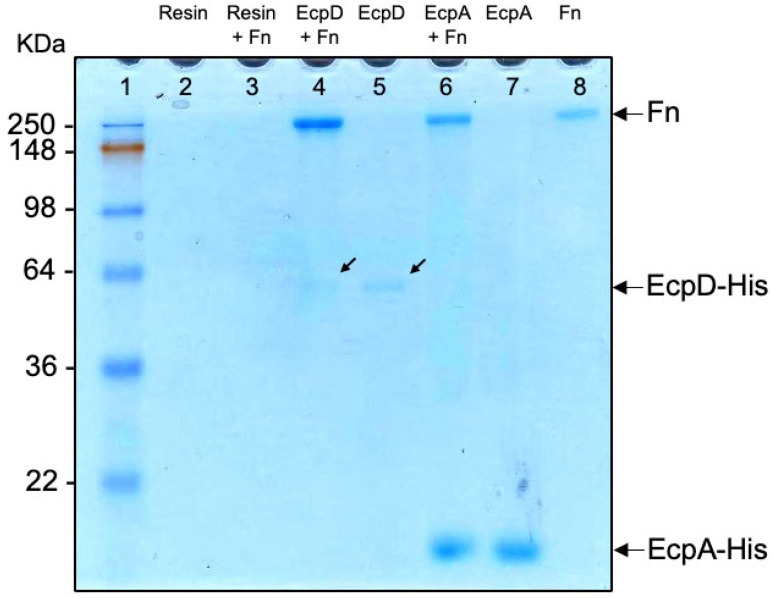
Binding of Ecp proteins to soluble Fn (pulldown assay). His-tagged Ecp proteins (10 μg/mL) were bound to nickel–NTA resin and then incubated for 1 h with 100 μL Fn (50 μg/mL) at room temperature. The resin was washed three times with PBS by centrifugation, resolved in a 10% SDS-PAGE gel, and stained with a Coomassie blue dye. Samples containing only resin (lane 2) and resin plus Fn (lane 3) were worked in parallel as controls. Individual proteins EcpD (lane 5), EcpA (lane 7), and Fn (lane 8) were electrophoresed and used as mass controls. Lane 1, molecular mass standards. The arrows point to EcpD.

For this set of experiments, His-tagged Ecp proteins (10 μg/mL) were first bound to nickel–NTA resin, and the washed resin was then incubated for 1 h with 100 μL Fn (50 μg/mL) at room temperature. The resin was washed three times with PBS by centrifugation and resolved in 10% SDS-PAGE gels. Analysis of the Coomassie blue-stained gels shows that both EcpD and EcpA proteins bind to Fn (Figure 8).

## 3. Discussion

ECM glycoproteins connect the extracellular components with the internal signaling machinery, so that signal transduction regulates the gene transcription of structural proteins and integral cellular processes. ECM glycoproteins have been demonstrated as receptors for several bacterial pathogens. Fn-binding proteins have been described in numerous intestinal pathogens including *Salmonella* Typhimurium, *Yersinia pseudotuberculosis* and *Y. enterocolitica*, *Pseudomonas aeruginosa*, and several *E. coli* pathotypes [16,27]. Surface-associated bacterial proteins such as outer membrane proteins, curli, and flagella have been shown to recognize Fn, Ln, vitronectin, and/or Cn [9,28]. Non-intestinal pathogens such as *Brucella abortus*, *S. aureus*, *N. gonorrhoeae*, and *Haemophilus influenzae* have also been shown to display affinity for basal membrane proteins [15,19,20,29] as part of their host-cell binding scheme.

The interaction of EPEC with the intestinal epithelium is a multifaceted interplay that involves numerous adhesins that act synergistically to successfully achieve host colonization [30,31,32]. The ECP is an *E. coli* surface structure that promotes biofilm formation and cell adherence [13]. The receptors recognized by the ECP on epithelial cells are unknown. In this study, we investigated the possibility of recognition of ECM glycoproteins by the ECP. We showed that EPEC O127:H6 prototypic strain E2348/69 binds to ECM glycoproteins, including Fn, Ln, Cn-I/IV, and Mu, through the ECP tip adhesin EcpD. In vitro assays comparing the binding of Δ*ecpA* and Δ*ecpD* isogenic mutants to the wild-type strain as well as using recombinant His-tagged EcpA and EcpD proteins to ECM glycoproteins were performed. Our investigation reveals that the ECP of EPEC E2348/69 binds to several ECM glycoproteins with different affinity as determined in an ELISA-based binding assay. In particular, the binding was significantly reduced in the Δ*ecpD* mutant, which totally lacks ECP. The Δ*ecpA* mutant was also reduced in binding but to a smaller extent than the Δ*ecpD* mutant. One explanation of the reduced binding of the Δ*ecpA* mutant is that although it displays EcpD on the surface, the lack of the EcpA-containing fiber reduced hydrophobicity of the strain, affecting bacterial interaction with ECM glycoproteins. Furthermore, the binding of EPEC strains to either immobilized or soluble Fn or to Ln was assessed by immunofluorescence or immunoelectron microscopy. These immunoassays confirmed previous EPEC–ECM glycoproteins binding patterns. All together, these data suggested a role for EcpD as a tip adhesin and in host-cell receptor recognition.

In a different set of experiments employing purified recombinant EcpA and EcpD proteins to test their ability to bind to immobilized ECM glycoproteins, we found that both proteins bound to Ln, Fn, collagens, and Mu in a dose-dependent fashion, although EcpD yielded a greater binding affinity for all the conditions tested when compared to EcpA. Differences in the binding affinity between the native proteins on the bacteria versus the purified ECP proteins can be explained by the possible loss of binding sites determined by conformational structure. However, the overall trends were preserved between these two different approaches.

It is possible that the ECP of EPEC is responsible for binding ECM glycoproteins in the basal lamina, contributing to increased inflammation in the small bowel if the external matrix is disturbed. This could stimulate proinflammatory cytokines, leading to the recruitment of immune cells, perhaps damaging host tissues. EPEC infection is characterized by the formation of AE lesions, in which the intercellular tight junctions are altered by T3SS effectors, conceivably exposing components of the basal membrane to EPEC [3,33]. It is tempting to speculate that the presence of ECM glycoproteins contributes to the adherence of EPEC to the mucosal epithelium by generating a stronger binding interaction during gut colonization. EcpD may not be the only EPEC component recognizing ECM glycoproteins since the Δ*ecpD* mutant still retained a considerable binding activity. In sum, the EPEC ECP-mediated binding to ECM glycoproteins is determined by the presence of the tip-associated protein EcpD and, to a smaller extent, by the EcpA major pilin subunit. It is important to note that all the pathogenic and nonpathogenic *E. coli* produce ECP, which implicate EcpD as an important adhesin in all the *E. coli*, allowing different strains to adapt to different hosts and niches.

## 4. Materials and Methods

### 4.1. Bacterial Strains, Plasmids, and Growth Conditions

Strains and plasmids used in this study are listed in Table 1. Nonpolar Δ*ecpA* and Δ*ecpD* mutants derived from E2348/69 were previously characterized genetically and phenotypically and were available from previous studies [11,13,14]. For routine work, bacterial strains were grown in the Luria–Bertani (LB) (Sigma) broth at 37 °C with shaking. For induction of the ECP, the strains were cultured at 26 °C in Dulbecco’s modified Eagle’s medium (DMEM) (Invitrogen). When necessary, antibiotics at concentrations of 50 µg/mL (kanamycin) and 30 µg/mL (chloramphenicol) were added. L-(+)-arabinose (Sigma) was used at a final concentration of 100 mM to induce the expression of the lambda Red recombinase system from plasmid pKD46 [34].

### 4.2. ECM Glycoproteins

Fibronectin (Fn) (from bovine plasma), laminin (Ln) (Engelbreth–Holm–Swarm murine sarcoma), collagens I and IV (Cn-I and Cn-IV), mucin (Mu) (type I-S bovine submaxillary glands), and bovine albumin serum (BSA) were purchased from Sigma (St. Louis, MO, USA).

### 4.3. Antibodies

For the various binding assays performed, we used highly absorbed rabbit antibodies specific for ECP, EcpA, and EcpD, which were available from previous studies [11,13,14]. Anti-His antibodies were obtained from Sigma. Anti-O127 serum was from BD Difco (Franklin Lakes, NJ, USA).

### 4.4. Bacterial Binding to ECM Glycoproteins

ECM glycoproteins (Fn, Ln, Cn-I, and Cn-IV) and Mu were applied at a concentration of 10 mg/mL/well in triplicate to ELISA plates in a coating carbonate buffer (pH 9.8) at 4 °C for 18 h. Then, the plates were washed five times with PBS–0.01% Tween (PBST) and 1 × 10^8^ bacteria in 100 μL of bacterial cultures (wild-type EPEC strain E2348/69, Δ*ecpA* or Δ*ecpD* mutants) were added for 1 h at room temperature. After washing three times with PBST, an antibody against LPS O127 was added at 1:3000 dilution for 1 h followed by a washing step and the addition of an anti-rabbit IgG–peroxidase conjugate (1:3000) for an additional hour. After washing, the peroxidase substrate (Sigma) was added and the color developed was read at 595 nm using a spectrophotometer. In addition, colony-forming units (CFUs) were also quantitated from replica plates. BSA was used as a negative control.

### 4.5. Immunofluorescence

A droplet of Fn (10 µg/mL) was applied onto glass coverslips and let to air-dry, fixed with 2% formalin, and blocked with 1% BSA. Ten μL of a bacterial suspension (1 × 10^9^ bacteria/mL) of E2348/69, Δ*ecpA* and Δ*ecpD* mutant strains were added for 1 h and then washed three times. Binding between bacteria and Fn was determined by immunofluorescence using primary anti-O127 antibodies followed by the secondary Alexa Fluor 488 conjugate. The samples were then examined using an Axio Imager 1.0 Zeiss microscope.

### 4.6. Electron Microscopy Studies

To assess binding of Fn to EPEC strains, we incubated DMEM-grown bacteria in the presence of Fn (10 mg/mL) for 1 h at room temperature. After washing with PBS, the bacteria were spotted on a 300-mesh copper electron microscopy grid and reacted with commercial rabbit anti-Fn antibody (Sigma) (1:10 dilution in 1% horse serum/PBS) for 1 h followed by incubation with an anti-rabbit IgG–10 nm gold conjugate (BBI) (1:10 dilution in 1% horse serum/PBS). The bacteria were then stained with 1% phosphotungstic acid (pH 7.2) for 2 min and observed using a JEOL electron microscope. To immunostain the pili, E2348/69 was incubated with rabbit anti-EcpA and/or anti-EcpD antibodies and suitable 10 nm and 20 nm gold conjugates.

### 4.7. Inhibition of Bacterial Binding to Fn

The wells of the ELISA plates were coated overnight with 10 µg/mL of Fn. The wells were washed three times with PBST and blocked with 3% BSA. Wild-type EPEC strain E2348/69 and the respective mutants were grown in DMEM at 26 °C, washed, and adjusted to an OD of 1.0 at 600 nm. Approximately 1 × 10^8^ bacteria in 100 μL were preincubated with Fn (10 or 50 mg/mL) for 1 h, and the mixture was then added to the Fn-coated wells for 1 h. After washing, anti-O127 antibodies (1:1000) were added for 1 h followed by the addition of an anti-rabbit IgG–peroxidase conjugate. A peroxidase TMB substrate solution (KPL) was added and the development of color was read at 595 nm.

### 4.8. Binding of Recombinant EcpA and EcpD Proteins to Host Proteins

The His-tagged EcpA and EcpD were purified from laboratory *E. coli* BL21 harboring the plasmids pecpa-MH (EcpA-His) and pDB5-EcpD (EcpD-His) (Table 1) [11] and purified in a nickel column as recommended by the manufacturer (Qiagen). The purified proteins were biotinylated for further studies following the manufacturer’s protocol (Pierce). To determine if the aforementioned ECP proteins were capable of binding to ECM glycoproteins, an ELISA-based binding assay was designed, in which 96-well ELISA plates were coated overnight with various concentrations of Fn, Ln, and Cn-IV in carbonate buffer (pH 9.8). BSA was used as a negative control. Biotinylated EcpA and EcpD proteins were added at 25 µg/mL/well to ELISA plates for 1 h at room temperature. After washing three times with PBST, a streptavidin–peroxidase conjugate was added for 1 h and the OD was detected by the addition of a TMB substrate solution (KPL) at 595 nm.

### 4.9. Dose-Dependent Binding of ECM Glycoproteins to EcpA and EcpD Proteins

To determine the dose-dependent binding of EcpA and EcpD proteins, 10-fold dilutions (ranging from 0.1 to 100 µg/mL) of biotinylated EcpA or EcpD were added in quadruplicate for 1 h after blocking of the plates with 3% BSA. Unbound proteins were removed by washing followed by 1 h incubation with absorbed anti-ECP serum (1:1000). An anti-rabbit IgG–streptavidin–peroxidase conjugate was used as the secondary antibody (1:500) before the addition of the peroxidase substrate. The experiments were performed at least three times on separate days, and the data are expressed as the means of the averages of the results obtained from the experiments performed.

### 4.10. Inhibition of Binding of Ecp Proteins to Fn

First, ELISA plates were coated overnight with fibronectin (10 mg/mL). The plates were washed and blocked with 3% BSA for 1 h at room temperature. Separately, 25 µg/mL of biotinylated EcpA or EcpD proteins were preincubated with 10 and 50 µg/mL of Fn for 1 h, and then the mixture was added to the wells. After washing three times with PBST, a streptavidin–peroxidase conjugate was added for 1 h to detect bound biotinylated proteins. Finally, a TMB substrate solution (KPL) was added, and the colorimetric reaction was read at 595 nm.

### 4.11. Pulldown Assay

This experiment was performed to assay the binding of soluble Fn to EcpA-His or EcpD-His immobilized onto a resin bed. Ten µg/mL of recombinant His-tagged EcpA and EcpD were incubated overnight with Ni–NTA agarose resin (Qiagen). The following day, the mixtures were centrifuged and the pellets were washed three times with PBS to remove excess unbound proteins. Then, 100 µL of Fn (50 µg/mL) were added and incubated for 1 h at room temperature. In parallel, tubes containing only resin or resin plus Fn were included as controls. To remove unbound Fn, the mixtures were centrifuged and washed three times with PBS. The resin pellets containing EcpA-Fn or EcpD-Fn were resuspended in PBS, separated in a 10% SDS-PAGE gel, and stained with a Coomassie blue dye. Individual His-tagged EcpA or EcpD proteins were also electrophoresed as controls. The presence of two proteins per reaction in the Coomassie-stained gel indicated interaction between them.

### 4.12. Statistical Analysis

Quantitative assays were carried out three times in triplicate on different days. Standard deviations are represented by error bars and the data represent the average of all the results obtained from the three experiments performed. To compare two groups, a statistical analysis was completed using the nonparametric Mann–Whitney U test. When three or more groups were compared, data were analyzed using the nonparametric Kruskal–Wallis test. The level of significance was set at a *p*-value < 0.05 for all comparisons. The GraphPad Prism 9 software (GraphPad, San Diego, CA, USA) was used.

## Figures and Tables

**Table 1:** List of the strains and plasmids used.

Strains	Characteristics	Reference or Source
E2348/69	O126:H6 (wild-type prototype) Sm^r^	[35]
E2348/69 Δ*ecpA*	Wild-type strain mutated in *ecpA*::Km	[13]
E2348/69 Δe*cpD*	Wild-type strain mutated in *ecpD*::Cm	[11]
*E. coli* K12 DH5α	Laboratory strain	ATCC
**Plasmids**		
pKD46	Red recombinase system plasmid	[34]
pKD4	Km cassette template plasmid	[34]
pKD3	Cm cassette template plasmid	[34]
pBAD-MycHis A	Expression vector	Invitrogen
pET-28a	Expression vector	Millipore-Sigma
pecpa-MH	*ecpA* cloned into pBAD-MycHis A	[11]
pDB38	*ecpD* cloned into pET-28a	Lab collection

## Data Availability

Not applicable.
